# Is it the end of TILLING era in plant science?

**DOI:** 10.3389/fpls.2023.1160695

**Published:** 2023-08-22

**Authors:** Miriam Szurman-Zubrzycka, Marzena Kurowska, Bradley J. Till, Iwona Szarejko

**Affiliations:** ^1^ Institute of Biology, Biotechnology and Environmental Protection, Faculty of Natural Sciences, University of Silesia in Katowice, Katowice, Poland; ^2^ Veterinary Genetics Laboratory, University of California, Davis, Davis, United States

**Keywords:** TILLING, mutagenesis, crops, reverse genetics, functional genomics, mutations, plants, NGTs

## Abstract

Since its introduction in 2000, the TILLING strategy has been widely used in plant research to create novel genetic diversity. TILLING is based on chemical or physical mutagenesis followed by the rapid identification of mutations within genes of interest. TILLING mutants may be used for functional analysis of genes and being nontransgenic, they may be directly used in pre-breeding programs. Nevertheless, classical mutagenesis is a random process, giving rise to mutations all over the genome. Therefore TILLING mutants carry background mutations, some of which may affect the phenotype and should be eliminated, which is often time-consuming. Recently, new strategies of targeted genome editing, including CRISPR/Cas9-based methods, have been developed and optimized for many plant species. These methods precisely target only genes of interest and produce very few off-targets. Thus, the question arises: is it the end of TILLING era in plant studies? In this review, we recap the basics of the TILLING strategy, summarize the current status of plant TILLING research and present recent TILLING achievements. Based on these reports, we conclude that TILLING still plays an important role in plant research as a valuable tool for generating genetic variation for genomics and breeding projects.

## 1 What is TILLING and how to create a TILLING platform?

Mutation induction through chemical or physical mutagenesis has long been used in plant breeding as it creates new alleles and can lead to the development of new, agronomically important traits (for further reading see Maluszynski et al., 2017). It is also used in genetic studies for functional analysis of mutated genes. There are two main approaches to such studies: “forward” and “reverse”. The classical forward approach starts with the identification of an interesting phenotype and then the mutated gene responsible for this phenotype can be searched for, usually through mapping and positional cloning. The “reverse” approach is the other way around – first, the mutation within the gene of interest is identified and afterward the phenotype caused by this mutation is analyzed (Griffiths et al., 2004).

**Figure 1:**
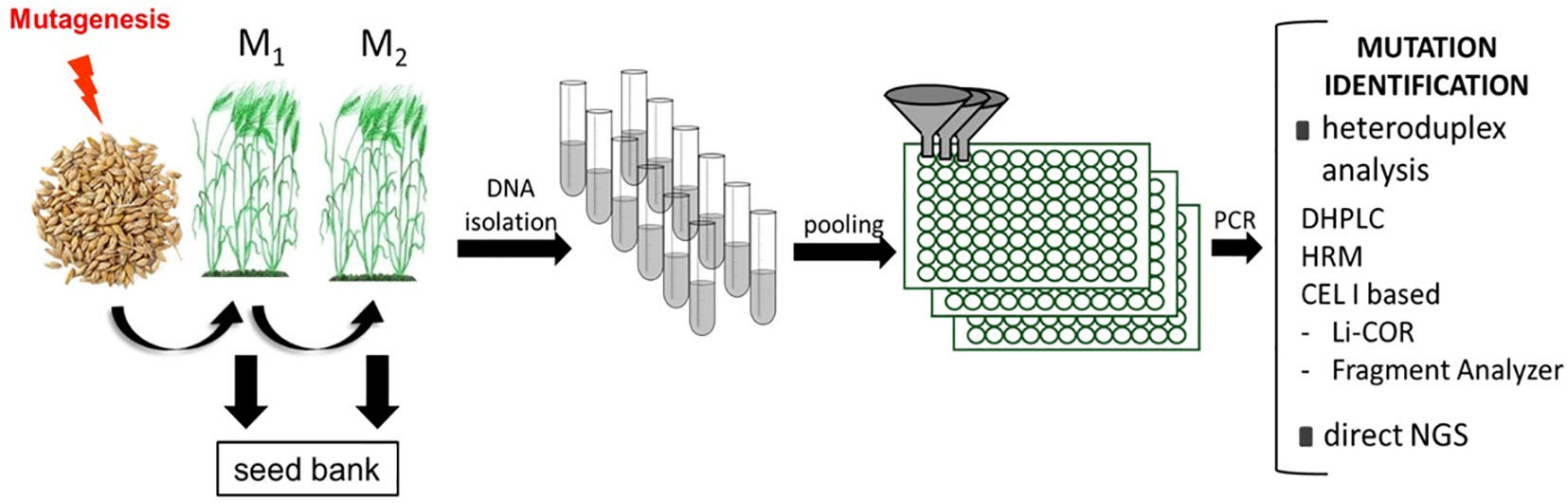
The general flow of traditional TILLING strategy. The first step is the mutagenesis of seeds. The seeds are propagated into further generations and DNA is isolated from M_2_ plants individually. The next step is DNA pooling, followed by the PCR reactions for specific amplicons. There are many methods of mutation identification - either direct or based on heteroduplex analysis. After the identification of a particular plant carrying a mutation in a gene of interest, the phenotypic evaluation should be performed to assign the gene function. DHPLC, Denaturing High-Performance Liquid Chromatography; HRM, High-Resolution Melting; CEL I- endonuclease extracted from a Celery Juice Extract; NGS, Next Generation Sequencing.

TILLING (Targeting Induced Local Lesion IN Genomes) was first developed for *Arabidopsis thaliana* as an alternative to insertional mutagenesis. It was described as a reverse genetic strategy that combines traditional random chemical mutagenesis with rapid mutational screening for induced lesions in genes of interest (McCallum et al., 2000a; McCallum et al., 2000b). However, once established, a TILLING population may be used for forward genetic studies as well. Immediately after its invention, TILLING has been adapted and deployed for large-scale screening of induced mutations not only in Arabidopsis but also in other plants (reviewed in Colbert et al., 2001; Henikoff et al., 2004; Stemple, 2004). At the beginning of TILLING, one of the biggest limitations of this technology was the necessity of knowing the sequence of the gene of interest (or at least its fragment) and TILLING relied on the emergence of practical genome sequence tools. However, as more and more plant species have been fully sequenced in recent years and there is an enormous amount of sequence data and bioinformatic tools for their analysis, these issues are becoming less limiting. Additionally, throughout the years TILLING is being continuously modified to make it more robust, especially in terms of mutation identification (discussed in the next section). The general scheme of TILLING strategy is described in detail in Szarejko et al, 2017 and Szurman-Zubrzycka et al., 2017, and illustrated briefly in **Figure 1**.

The goal of mutagenesis for TILLING is to obtain a highly mutagenized population to increase the probability of finding a mutation in every gene. Thus, it is crucial to select a proper mutagen and its dose. The sensitivity for mutagen treatment varies between plant species and even between various genotypes of the same species. In general, the lower the mutation frequency, the larger population is needed, which leads to higher costs and labor efforts. On the other hand, too high mutation frequency may cause lethality or sterility of M_1_ plants. The most popular mutagen used for the creation of TILLING populations is EMS (Ethyl methanesulphonate) which is an alkylating agent causing mainly G/C to A/T transitions (Kurowska et al., 2011). The mutation density in different TILLING populations is very diverse and ranges from ~1/7 Mb to 1/20 kb (**Table 1**; Till et al., 2018). Once the dose of a mutagen is optimized, the TILLING platform may be created. For generatively propagated crops, the material to be mutagenized is usually the seeds (Jankowicz-Cieslak et al., 2011). This is the best choice because seed mutagenesis is technically easy, there are no temporal or developmental stage restrictions for treatment, relatively high mutagen doses can be applied to dormant seeds, and they are easy to store and transport. Only mutations induced in generative line cells in the seed embryo are inherited, hence all molecular analyses aimed at the identification of induced mutations should be performed starting with the M_2_ generation. In regard to vegetatively propagated crops, such as banana (*Musa paradisica* L.), peppermint (*Mentha* L.) or sugarcane (*Saccharum officinarum*), the generation of stable mutants is more problematic. The meristematic buds (e.g. tubers, bulbs, rhizomes) or cell or tissue cultures *in vitro* are usually target materials for mutagenesis (Jankowicz-Cieslak et al., 2012; Jankowicz-Cieslak and Till, 2016). The chimera dissociation usually has to be performed e.g. by adventitious regeneration from a single somatic cell or by somatic embryogenesis (Geier, 2012). For generatively propagated plants, only one seed from the M_1_ plant should be propagated into the next generation (M_2_) in order to avoid the repetitions of the same mutations during screening. A very important aspect is the maintenance of mutagenized generations – each M_2_ plant and its M_3_ progeny should be assigned a unique code and seeds from each plant should be collected separately.

**Table 1:** Representative TILLING platforms for diploid and polyploid plant species.

Ploidy	Species	Cultivar/ landrace	Mutagen	Total size of M_2_ population	Mutations frequency	Number of screened genes	Methods of mutations identification	Year of platform creation and research conducted	References	Examples of other research with this platform
Diploid	Arabidopsis (*A. thaliana*)	Columbia-0	EMS	6,912	1/170 kb	100	CELI, LI-COR sequencer	2003	Till et al., 2003	
	Barley (*Hordeum vulgare*)	Optic	EMS	9,216	1/614 kb	2	CELI, Transgenomic WAVE-HS	2004	Caldwell et al., 2004	
		Morex	NaN_3_	4,906	1/374 kb	4	Surveyor^®^Mutation Detection Kit, LI-COR sequencer	2008 TILLMore	Talamè et al., 2008	Sparla et al., 2014; Kirschner et al., 2021; Fusi et al., 2022
		Lux	NaN_3_	9,575	1/2.5 Mb	2	CELI, gel-based, ABI PRISM^®^ 377 DNA Sequencer	2009	Lababidi et al., 2009	
		Barke	EMS	10,279	1/500 kb	6	CELI, LI-COR sequencer	2009	Gottwald et al., 2009	Li et al., 2021
		DH line ‘H930-36’	MNU; gamma rays	1,372; 1,753	1/504 kb 1/3207 kb	2	CELI, LI-COR sequencer	2012	Kurowska et al., 2012	
		Sebastian	Double treatment: NaN_3_ and MNU	9,600	1/477 kb	37	CELI, LI-COR sequencer	2018 *Hor*TILLUS	Szurman-Zubrzycka et al., 2018	Stolarek et al., 2015a; Stolarek et al., 2015b; Gruszka et al., 2016; Janeczko et al., 2016; Marzec et al., 2016; Mendiondo et al., 2016; Daszkowska-Golec et al., 2017; Daszkowska-Golec et al., 2018; Tramontano et al., 2019; Szurman-Zubrzycka et al., 2019a; Szurman-Zubrzycka et al., 2019b; Collin et al., 2020; Daszkowska-Golec et al., 2020; Jaskowiak et al., 2020; Marzec et al., 2020;
		Golden Promise	Twice mutagenized with EMS (M_0_ and M_2_)	3,072	1/154 kb	Targeted exon capture of 46 genes	Whole exome capture sequencing, validated by Sanger sequencing	2019	Schreiber et al., 2019	
		Hatiexi (HTX)	EMS	8,525	1/454 kb	56	Multiplexed amplicon sequencing	2022	Jiang et al., 2022	
					1/256 kb genome-wide	Genome region mapped - 3.8 Gb in each plants	Whole genome sequening			
					1/306 kb	1	CELI digestion and capillary electrophoresis			
	Lotus (*Lotus japonicus)*	Ecotype B-129 Gifu	EMS	4,904	1/502 kb	10	CELI, LI-COR sequencer	2003	Perry et al., 2003	Perry et al., 2009
	Maize (*Zea mays*)	B73 N3	EMS	384 366	1/485 kb	11	CELI, LI-COR sequencer	2004	Till et al., 2004	
	Melon (*Cucumis melo)*	Inbred line CharMono	EMS	4,023	1/573 kb	11	CELI, LI-COR sequencer	2010	Dahmani-Mardas et al., 2010	
	Pea (*Pisum sativum)*	Caméor	EMS	4,704	1/84 kb - 1/356 kb	20	CELI, LI-COR sequencer	2008	Dalmais et al., 2008	
	Rice (*Oryza sativa*)	Nipponbare	NaN_3_-MNU	768	1/265 kb	10	CELI, LI-COR sequencer	2007	Till et al., 2007	
		Dongan	Gamma rays	2,961	1/492 kb	9	CELI, ABI 3130XL capillary electrophoresis	2017	Hwang et al., 2017	
		Japonica, line DS552	Gamma rays	4,560	1/690 kb	2	High-resolution melting (HRM)	2018	Li et al., 2018	
	Sunflower (*Helianthus annuus* L.)	Inbred line GV342	EMS	3,651	1/475 kb	4	CELI, LI-COR sequencer	2011	Sabetta et al., 2011	
		Inbred line GV342	EMS	3,651	1/7,424 kb	21	Amplicon sequencing	2011 2021	Sabetta et al., 2011; Fanelli et al., 2021	
	Sorghum (*Sorghum bicolor* (L.) Moench)	BTx623	EMS	1,600	1/526 kb	4	CELI, LI-COR sequencer	2008	Xin et al., 2008	
	Soybean (*Glycine max* L. Merr.)	Forrest,	EMS	529	1/140 kb	7	CELI, LI-COR sequencer	2008	Cooper et al., 2008;	
		Williams 82	EMS	768	1/550 kb					
		Williams 82	MNU	768	1/140 kb					
		Forrest	EMS	4,032	1/127 kb	138	Target capture sequencing technology	2021	Lakhssassi et al., 2021b	Lakhssassi et al., 2021a
	Tomato (*Solanum lycopersicum* L.)	Tpaadasu	EMS	8,255	1/737 kb	5	Conformation sensitive capillary electrophoresis (CSCE), high- resolution melting (HRM)	2009	Gady et al., 2009	
		Red Setter	EMS	4,741 1,926	1/322 kb 1/574 kb	7 7	ENDOI, LI-COR sequencer	2010	Minoia et al., 2010	
		M82	EMS	4,759	1/574 kb	19	ENDOI, LI-COR sequencer	2010	Piron et al., 2010	Brisou et al., 2021
		Micro-Tom, dwarf tomato	EMS, gamma rays	3,052	1/237 kb	10	ENDOI, LI-COR sequencer	2011, University of Tsukuba, Micro-Tom	Okabe et al., 2011	Okabe et al., 2012; Okabe et al., 2013; Mubarok et al., 2019; Takei et al., 2019; Yan et al., 2021
		Micro-Tom, dwarf tomato	EMS, gamma rays	3,052	1/2 kb (mutation candidates)	All exomes	Multiplex exome sequencing	2011 2019	Yano et al., 2019; Okabe et al., 2011	
	Yellow mustard (yellow sarson) (*Brassica rapa s*ubsp. *trilocularis*)	Inbred line R-o-19	EMS	9,216	1/60 kb	6	CELI, ABI3730 sequencer	2010	Stephenson et al., 2010	Alcock et al., 2021
Polyploid	Oat (*Avena sativa*) (6x)	Bekinda	EMS	2,600	1/20-40 kb	2	RAPD-PCR fingerprinting, MALDI-TOF, DNA sequencing	2010	Chawade et al., 2010	
	Oilseed rape (*Brassica napus*) (4x)	Semi-winter rapeseed cv. Ningyou7	EMS	4,859	1/41.5 kb -1/130.8 kb	1	CELI, agarose gel	2008	Wang et al. 2008	
		Spring line YN01-429; the winter cultivar Express 617	EMS	5,361; 3,488	1/27 kb – 1/60 kb; 1/12 kb – 1/22 kb	2	CELI, LI-COR sequencer	2012	Harloff et al., 2012	
		Inbred line Express 617	EMS	7,680	1/15 kb - 1/64 kb	6 gene families (12 genes)	Amplicon sequencing (amplicon-seq)	2012 2020	Sashidhar et al., 2020; Guo et al., 2014	Elahi et al., 2015; Braatz et al., 2018
		Inbred line Express 617	EMS	3,488	1/24 kb - 1/72 kb	3	CELI, LI-COR sequencer	2012 2014	Guo et al., 2014; Harloff et al., 2012	
	Peanut (*Arachis hypogaea* L.) (4x)	Tifrunner	EMS	3,420	1/967 kb	Homologues of 3 (6 genes)	CELI nuclease digestion, and electrophoretic detection of labeled fragments	2011	Knoll et al., 2011	
		NC-7, Virginia market type	EMS	1,541	1/2139 kb	Homologues of 2 (4 genes)	Mutation discovery kit DNF-910-K1000T; AATI, Ames, USA,capillary electrophoresis (Fragment Analyzer™)	2021	Karaman et al., 2021	
	Tabacco (*Nicotiana tabacum*) (4x)	Yunyan 87	EMS	1,536	1/106 kb	7	CELI, AdvanCE FS96 system electrophoresis	2020	Gao et al., 2020	Taylor and Greene, 2003; Song et al., 2020
	Wheat (*Triticum aestivum (*6x*), T. turgidum (*4x)	Express (6x)	EMS	1,152	1/24 kb	Homoloques of one gene	CELI, LI-COR sequencer	2005	Slade et al., 2005	Slade et al., 2012; Avni et al., 2014
		Kronos (4x)		768	1/40 kb					
		Breeding line ‘UC1041+Gpc-B1/Yr36’ (6x)	EMS	1,536	1/38 kb	3	CELI, LI-COR sequencer	2009	Uauy et al., 2009	
		Kronos (4x)			1/51 kb	2				

NaN_3_, sodium azide; MNU, N-Methyl-N-NitrosoUrea; EMS, Ethyl MethaneSulfonate.

The mutation screening is performed on the M_2_ generation, because of the chimerism of the M_1_ generation owing to the fact that seeds are multi-cellular and only a subset of the mutagenized cells are responsible for germline development. The DNA should be isolated from each M_2_ plant individually (one M_2_ plant per M_1_ parent) and is usually pooled prior to screening for mutations. There are many screening methods aimed at mutation identification - they differ in sensitivity, capacity, difficulty, and costs. These methods are briefly described in the following section. Depending on the methods of mutation screening used, there is a different schema of DNA sample pooling. The M_2_ plants (and further generation) may be also directly phenotypically characterized for a forward genetic approach. Importantly, the proper maintenance of seeds from all generations (in a seed bank) and DNA stocks isolated individually from M_2_ plants are crucial to make the TILLING platform long-lasting. In order to make a TILLING platform publicly available, it is indispensable to create a database where all data about phenotypes, mutations identified and seed availability is collected.

The first TILLING population was developed for *Arabidopsis thaliana* in 2000. Because TILLING is a method that can be easily applied to most organisms, shortly afterwards new TILLING populations for other plant species were produced. Examples of TILLING platforms created for various, diploid and polyploid, plant species are presented in **Table 1**.

## 2 Methods for identifying mutations

The discovery that mutations can be induced revolutionized plant breeding. The first crop mutant variety (‘Chlorina’ – a variety of tobacco) was introduced in the 1930s (Tollenaar, 1934; Tollenaar, 1938) and methods of mutation induction with the use of physical or chemical mutagens have long been established for many plant species. In the TILLING approach, mutation induction is followed by mutation identification in the genes of interest. The detection of mutations at the DNA base level became possible with the development of sequencing methods beginning in the 1970s. In recent years DNA sequencing methodologies have experienced a renaissance, with methods continually evolving. This is resulting in higher throughput and lower costs for mutation discovery and other applications.

In the first TILLING experiments performed on *Arabidopsis thaliana* the method of point mutation identification was based on DHPLC (Denaturing High-Performance Liquid Chromatography) (McCallum et al., 2000a; McCallum et al., 2000b). Arabidopsis seeds were treated with EMS, DNA was isolated from M_2_ individuals and pooled, PCR reactions for selected gene regions were performed and after denaturation and annealing (allowing heteroduplex formation in the case of mutation appearance) the DHPLC method was used. This method allows the detection of heteroduplex in a pool as an additional peak in the chromatogram, because of differential melting kinetics of homo- and heteroduplexes of DNA. After the identification of the potential mutations in particular M_2_ plants, the mutant PCR products were sequenced to confirm the mutation. The DHPCL method is very sensitive, however, it is relatively low throughput. Another method used for direct analysis of heteroduplex appearance is HRM (High-Resolution Melting) which is very fast but restricted only to fragments of max. 400 bp length (Gundry et al., 2003; Szurman-Zubrzycka et al., 2017). In order to increase the efficiency of mutation discovery, single-strand-specific nucleases began to be used (Colbert et al., 2001). The most commonly used endonuclease is CEL I which recognizes heteroduplexes and cuts them in a position of mismatch (position of induced mutation). The observation of cleaved products was typically performed with the use of LI-COR sequencers, where up to 768 samples may be analyzed on one gel, in the case of 8-fold DNA pooling (Colbert et al., 2001). For the first two decades of TILLING research, this was the most common method of mutation discovery. To reduce the cost of TILLING screening it is possible to visualize the products of digestion on an agarose gel, but this method is not very sensitive and some mutations may go unnoticed. Recently, capillary sequencers (e.g. AdvanceCE 96 FS or Fragment Analyzer) are also becoming the instruments of choice for the identification of heteroduplex cleavage products (Jost et al., 2019).

Currently, the rapid identification of all mutations present in the mutant genome may be done through the NGS approach (‘TILLING by Sequencing’), either directly or using different strategies of sample pooling (one-, two- or three-dimensional) (e.g. Tsai et al., 2011; Fanelli et al., 2021). The huge restriction for broad usage of NGS for TILLING is its cost, however, NGS is becoming noticeably cheaper over time. Another problem is the production of very large datasets for bioinformatic analysis. Nevertheless, the collection of genomic data from individual plants from mutagenized populations opens up new possibilities for TILLING approach. One can simply screen collected data for plants carrying mutations in a gene (regions) of interest and order the seeds from a developer of TILLING population (Krasileva et al, 2017). To reduce the size of obtained data and to minimalize the acquisition of unnecessary data, ‘TILLING by Sequencing’ may be restricted only to coding fragments of DNA, when performing exome capture instead of whole genome sequencing. NGS methods may be also applied to screen a set of pooled PCR amplicons instead of sequencing of whole genome/exome. There are many different NGS technologies described up till now, with Illumina being the most popular in TILLING projects. However, we can suppose that in the future the long-read sequencing technologies, such as PacBio and Oxford Nanopore sequencing, or new technology like Linked-Read (10xGenomics), will also be used in TILLING strategy. This can contribute to increasing sequencing accuracy or improving the balance between costs and depth of sample sequencing. The methods of mutation identification used in different TILLING projects are shown in **Table 1**. Recently, Wang et al. (2023) showed a very exciting example of how to boost wheat functional genomics using a mutant population of common winter wheat variety KN9204 generated by EMS treatment, similar to the majority of TILLING population, however, authors did not describe their strategy as TILLING. Studies aimed to obtain gene-indexed mutants in every coding gene in the wheat genome, which is a huge effort from one side, but offers great opportunities from the other. The use of NGS technology, specifically exome capture sequencing of 2,090 mutant lines revealed that for almost all coding genes (99%) this goal was achieved. Potentially, it might be also the direction of progress in the analysis of TILLING population in other plant species.

Another new approach used to screen mutagenized populations is called FIND-IT (Fast Identification of Nucleotide variants by droplet DigITal PCR) (Knudsen et al., 2022). It provides ultrafast screening (10 days) for targeted genetic variants of interest (at single-nucleotide resolution). FIND-IT combines large-scale sample pooling with highly sensitive droplet digital PCR (ddPCR) for genotyping. It was efficiently validated for barley by fast screening of variant libraries from 500,000 individuals and isolating more than 125 targeted gene knockouts and other interesting variants (Knudsen et al., 2022).

## 3 Current status of TILLING in plant research

Even though the first TILLING service was developed more than 20 years ago, this strategy is still broadly used in plant research studies, especially for agronomically important species. TILLING mutants are used to study the genetic bases of many important traits related to plant development and response to various biotic and abiotic stresses. TILLING is also used to create genotypes with improved important agronomic traits e.g. related to climate change, and hence it holds great promise for addressing global challenges in agriculture and food security and may be helpful in achieving some of the Sustainable Development Goals (SDGs) adopted by the United Nations General Assembly in 2015 as part of the 2030 Agenda for Sustainable Development (https://sdgs.un.org). The newest achievements generated through the TILLING approach are illustrated in **Table 2**, where we describe the goal of using TILLING in a particular study, a species and genotype used for TILLING screening, a gene and encoded protein analyzed, its biological function, the created mutants and their characteristics.

**Table 2:** The recent achievements of TILLING in basic studies of gene function and/or breeding of commercial cultivars.

Category	Goal	Species/cultivar	Gene(s)	Function	Investigated mutant(s)	Effect of mutation	References
	Increasing drought tolerance	Barley (*H. vulgare*), cv. Sebastian	*HvABI5*	ABA Insensitive 5, a basic leucine zipper (bZIP) transcription factor	*hvabi5.d* carrying a missense mutation	Increase tolerance to drought stress by better membrane protection, higher flavonoid content, and faster stomatal closure	Collin et al., 2020
			*HvCBP20*	Cap-Binding Protein 20 - small subunit of nuclear Cap-Binding Complex (nCBC)	*hvcbp20.ab* carrying a missense mutation	Increase tolerance to drought stress by lower permeability of epidermis (increased wax deposition)	Daszkowska-Golec et al., 2020
			*HvERA1*	Enhanced Response to ABA1, β-subunit of farnesyltransferase	*hvera1.b* carrying a missense mutation	Increase tolerance to drought stress by better photosynthesis performance and a higher leaf RWC; semi-dwarf phenotype and ABA-sensitivity during seed germination	Daszkowska-Golec et al., 2018
	Uncovering the role of strigolactones in response to drought		*HvD14*	Strigolactones receptor	*hvd14.d* carrying missense mutation	Hyper-sensitive to drought stress	Marzec et al., 2020
	Increasing plant tolerance to salinity	*Yellow mustard (Brassica rapassp. trilocularis)*, *Inbred line R-o-18 s*	*CAX1a*	Ca^2+^ cation exchangers (CAX) transporter	*BraA.cax1a-7, BraA.cax1a-4, BraA.cax1a-12* carrying missense mutations	*BraA.cax1a-4* provided higher biomass, better photosynthetic performance - higher water use efficiency, Fv/Fm, electron fluxes, and Rubisco values; mutants presented increased osmotic protection through a myo-inositol accumulation	Navarro-León et al., 2021
	Decreasing heavy metal accumulation	Tobacco (*N. tabacum*), Yunyan 87	*HMA2S HMA4T*	Heavy metal transporters (Cd and Zn)	Nonsense (*hma2s-7*) missense (*hma4t-3*)	The levels of Cd and Zn decreased in leaves which was confirmed by CRISPR/Cas9- knockout mutants	Gao et al., 2020
	Alleviating Zn deficiency and toxicity, improvement in Cd phytoremediation programs	Yellow mustard (*Brassica rapa* ssp. *trilocularis*), inbred line R-o-18	*HMA4a*	Heavy metal ATPase or Heavy Metal Associated (HMA), transporter which is able to transport Cd or Zn from the root to the shoot	Mutant carrying a missense mutation (*BraA.hma4a-3*), identified by Lochlainn et al., 2011	Higher tolerance to Cd and Zn	Blasco et al., 2019; Navarro-Leon et al., 2019a
	Increasing aluminium tolerance, functional analysis of ATR in barley	Barley (*H. vulgare*), cv. Sebastian	*HvATR*	Ataxia Telangiectasia and Rad-3-related protein kinase, involved in DDR pathway	*hvatr.g* and *hvatr.i* mutants carrying a missense mutation	Altered Aluminium response – accumulation of DNA damage; the frequency of cell division in roots not reduced after Al treatment	Szurman-Zubrzycka et al., 2019b
	Cold response, adaptation to chilling stress	Rice (*O. sativa*), japonica, cv.Zhonghua 11 (ZH11)	*OsCIPK7*	Calcineurin B-like interacting protein kinases	Three mutants (two with missense, and one with nonsense mutation)	Increased chilling tolerance (one mutant with missense mutation)	Zhang et al., 2019
	Improvement in the thermotolerance of tomato varieties	Tomato (*Solanum lycopersicum*), cv. Red Setter	*HSBP1*	Heat Shock Binding Protein 1	One mutant with a missense mutation, identified by Minoia et al., 2010	Enhanced basal thermotolerance, mature plants exhibit increased resilience under repeated HS treatments	Marko et al., 2019
Product quality	Improving seed oil composition - elevating oleic acid and lowering polyunsaturated fatty acid contents	Soybean (*Glycine max* L. Merr.), cv. Forrest	*GmSACPD-C* *GmFAD2-1A* *GmFAD2-1B* *GmFAD3A-C*	Fatty acid desaturases that control saturated/unsaturated fatty acid ratio	In total missense – 92 nonsense - 5	Fatty acid phenotypes of the 24 mutants have been improved (missense, nonsense)	Lakhssassi et al., 2021b
	Low phytic acid mutants identification	Oilseed rape (*Brassica napus*), inbred line Express 617	*Bn2-PGK*	2-phosphoglyceric acid kinase involved in the phytic acid synthesis pathway	Four mutants: two carrying mutations in a single gene, two carrying mutations in two paralogs	2-PGK2 (2-phosphoglyceric acid kinase) double mutants had significantly reduced phytic acid contents	Sashidhar et al., 2020
	Development of reduced-immunogenicity wheat genotypes relevant to most gluten-sensitive individuals with celiac disease	Common wheat (*T. aestivum*), cv. Express; Durum wheat (*T. durum*),cv. Kronos	*DME* *DRE2*	DEMETER, 5-methylcytosine DNA glycosylase/lyase causes demethylation; DRE2 an iron-sulfur cluster biogenesis enzyme	Double mutants in durum wheat and triple mutants in common wheat with complete DME or DRE2 activity suppression	Mutants displayed reduced content of immunogenic gluten proteins while retaining essential baking properties	Wen et al., 2022
	Elevating the levels of resistant starch, an important component of dietary fiber, associated with health benefits such as reduced glycaemic response	Common wheat (*Triticum aestivum*), cv. Cadenza	*SSIII*	Starch synthase III plays a key role in starch biosynthesis (synthesis of long amylopectin chains)	Triple *ssIIIa* mutants carrying mutations in each homoeologous copy of *ssIIIa* (A, B, and D)	Starch chain length distributions, increased levels of amylose, and fewer long amylopectin chains. Mutants, had more resistant starch and greater levels of non-starch polysaccharides	Fahy et al., 2022
	Increasing the resistant starch content (human health benefits)	Common wheat (*T. aestivum*); cv. Jagger, hard red winter cultivar	*SSIIa*	Starch Synthase	Triple null mutants	High amylose and resistant starch	Schoen et al., 2021
	Enrichment of provitamin A content in durum wheat grain	Durum wheat (*T. durum*), cv. Kronos	*HYD1*	β-carotene hydroxylase 1	Mutation in A and B subgenomes (a splice site, nonsense)	Increase of β-carotene by more than 70%	Garcia Molina et al., 2021
			*lcyE*	Lycopene ϵ-cyclase	Mutation in A and B subgenomes (nonsense, a splice site)	Increase in β-carotene content by roughly 75%	Sestili et al., 2019
	Development of non-transgenic glyphosate-tolerant wheat	Common wheat *(T. aestivum)*, cv. Express	*EPSPS*	Enzyme 5-enolpyruvylshiki- mate-3-phosphate synthase	Double mutant with missense mutations in A and D subgenome	Mutant exhibits substantial tolerance to commercially relevant levels of glyphosate	Moehs et al., 2021
	Investigating if CAX1 modifications have effects on plant metabolism	Yellow mustard (*Brassica rapa* ssp. *trilocularis*), inbred line R-o-18	*CAX1a*	Cation/H^+^ exchanger transporters	Three mutants carrying missense mutation, identified by Lochlainn et al., 2011	Inhibited some N metabolism enzymes, activated photorespiration activity, increased tolerance to high Ca^2+^	Navarro-Leon et al., 2019b
	Improvement of fruit shelf-life	Tomato (*Solanum lycopersicum*), line M82	*SlACO1* *SlE8*	ACC oxidase 1	In total 6 mutants (missense)	Mutants *slaco1-1* and *slaco1-2* showed decreased ethylene production and conductivity, enhanced shelf-life and firmness; *sle8-1* showed enhanced ethylene levels and reduced shelf-life, accelerate ripening	Brisou et al., 2021
	Increasing the grain size and weight	TILLING mutants were identified in durum wheat (*T. durum*), cv. Kronos. The mutations were introduced to common wheat (*T. aestivum*), cv. Paragon	*TaGW2*	RING-type E3 ubiquitin ligase takes part in the ubiquitin-proteasome pathway and regulates cell division	Seeds of the Paragon BC_1_ NIL (Near-isogenic lines) carrying the three TILLING-derived mutant alleles	Mutants carrying single-copy nonsense mutations in different genomes displayed an increase in GS and TGW. The enhanced effect was visible in the double and triple mutants.	Wang et al., 2018
	Investigating the role of SS4 in wheat	Durum wheat (*T. durum*), cv. Kronos	*SS4*	Glucosyltransferase, Starch Synthase 4	Mutation in A and B subgenomes (nonsense, a splice site)	Alterations in endosperm starch granule morphology; during early grain development, most amyloplasts in the mutant formed compound granules due to multiple initiations; reduced starch content in leaves and pollen grains	Hawkins et al., 2021
	Improvement of lignocellulosic quality of sorghum	Sorghum (*Sorghum bicolor*), line BTx623	*Bmr19 locus;* *FPGS gene*	Enzyme involved in folate (C1) metabolism, putative folylpolyglutamate synthase	Four mutants (missense), one with leaf Midrib phenotype (brown)	Reductions of the lignin content in the biomass, impair lignin biosynthesis.	Adeyanju et al., 2021
	Development of pea varieties lacking saponins in their seeds. that can impart an undesirable bitter taste	Pea (*Pisum sativum*), winter pea line Ps336/11 and Caméor spring pea	*PsBAS1*	β-amyrin Synthase 1	In Ps336/11 TILLING population: in total 8 mutations, one nonsense; in the Caméor TILLING population: in total 17 mutations: 3 intronic, 3 silent, 10 missense, 1 splice site	Two homozygous mutants seeds accumulated virtually no saponin, identified in two genetic backgrounds (Ps336/11, Caméor)	Vernoud et al., 2021
Other traits and processes	Examination of the role of wheat OMT2 in methylated flavone biosynthesis	Durum wheat (*Triticum turgidum* L), cv. Kronos	*OMT2*	O-methyltransferase catalyzes an O-methylation of the hydroxyl groups in flavones	Loss-of-function mutants of *OMT2* homoeologs (*omt-A2* and *omt-B2*); double mutant	Increased levels of chlorogenic acid in glumes of a mutant is suggesting that it might serve as a substrate for OMT2	Cain et al., 2022
	Functional analysis of DMC1 in barley involved in DNA repair	Barley (*H. vulgare*), cv. Sebastian	*HvDMC1*	Disrupted Meiotic cDNA1 recombinase that takes part in the repair of double-strand break (DSB)	*hvdmc1.c* carrying a missense mutation	Increased frequency of all chromosome aberrations during meiosis	Szurman-Zubrzycka et al., 2019a
	Generation of semi-dwarf and dwarf genotypes to study the relevance of brassinosteroids in barley development		*HvDWARF*	BR-6-oxidases, involved in brassinosteroids biosynthesis	Seven missense, one splice site	The short stature of various degrees and disturbance in brassinosteroids biosynthesis (mutation at a splice site led to dwarf phenotype, three missense to semi-dwarf mutants)	Gruszka et al., 2016
	Checking whether *NLP* genes in the *Triticeae* crops are involved in nitrate regulation and nitrogen use efficiency (NUE)	Barley (*Hordeum vulgare* L.), cv. Tamalpais	*HvNLP2*	Nodule Inception-like Protein that plays essential roles in nitrate signaling	*hvnlp2-1* carrying a missense mutation	Nitrate content is significantly higher, which may result from the decreased assimilation of nitrogen caused by reduced nitrate reductase activity and expression of nitrate assimilatory genes; mutants exhibited reduced biomass, seed yield, and NUE	Gao et al., 2022
	Investigating the role of OsERL in rice	Rice (*Oryza sativa* L.), Zhonghua 11 (ZH11)	*OsERL*	Leucine-rich repeat receptor-like kinase	In total 76 mutants, 19 missense, and 2 nonsense	Defects in the anther development, male sterility, or reduced numbers of anther lobes (surprisingly, two nonsense mutations led to a moderate effect, and two missense mutations led to a strong effect)	Liu et al., 2021

FAD2, delta(12)-fatty-acid desaturase; Ara h 1, vicilin; SACPD, delta-9-stearoyl-acyl carrier protein desaturase catalyzes the conversion of stearic acid to oleic acid; FAD2, the omega-6 fatty acid desaturase 2 converts oleic acid into linoleic acid; FAD3, the microsomal omega-3 fatty acid desaturase converts linoleic acid to linolenic acid.

### 3.1 TILLING for improved climate resilience

There is a threat that in the future we will face even more severe than now problems in terms of climate change and global warming. Extreme weather events such as heatwaves, drought, wildfires on the one hand, and flooding on the other, salinization of the soil, or violent weather phenomena like hurricanes, will happen more often. We are all witnesses of global climate change. It has been noticed that in the years 2011-2022 the temperature increased by 1.09°C (IPCC, 2022). The newest prediction presented by the sixth Assessment Report of the Intergovernmental Panel on Climate Change (IPCC, 2022) showed that in the 21^st^ century, we can expect the global mean temperature to increase between 1 to 5.7°C, depending on the scenarios, i.e. taking into account the level of greenhouse gas emission. Global warming already affects agriculture, and in the long term also food security. It has been estimated by a global-scale meta-analysis that one Celsius degree increase in global mean temperature will lead to a yield loss in four major crops: wheat (*Triticum aestivum* L., 6%), rice (*Oryza sativa* L, 3.2%), maize (*Zea mays*, 7.4%), and soybean (*Glycine max* L., 3.1%) (Zhao et al., 2017). The TILLING approach has been used to study response to abiotic stresses imposed by climate changes (drought, salinity, Al toxicity) in many important crops.

Drought belongs to abiotic stresses that affect crop productivity most strongly. Drought stress tolerance is a complex trait, therefore a great number and variety of genes can be selected for analysis by TILLING and both, drought-tolerant and drought-sensitive mutants have been created (Collin et al., 2020; Daszkowska-Golec et al., 2020; Marzec et al., 2020). In barley (*Hordeum vulgare* L.), a number of mutants in different genes encoding proteins engaged in response to drought e.g. bZIP transcription factor HvABI5 (ABA Insensitive 5), a protein involved in RNA metabolism - HvCBP20 (Cap-Binding Protein 20), or a subunit of farnesyltransferase HvERA1 (Enhanced Response to ABA1) have been developed. Some of induced alleles led to the increased tolerance to drought stress and influenced such physiological traits as stomata closure, relative water content (RWC), photosynthesis performance, membrane permeability, pigment content in leaves, and wax deposition (Daszkowska-Golec et al., 2018; Collin et al., 2020; Daszkowska-Golec et al., 2020). Tolerance to salinity, another severe abiotic stress in many agricultural regions, has also been studied using TILLING approach. The yellow mustard (*Brassica rapa ssp. trilocularis*) mutant carrying a substitution in a gene encoding Ca^2+^ cation exchanger transporter (CAX) showed a higher biomass, better photosynthetic performance, higher water use efficiency, and increased accumulation of a myo-inositol, which influenced osmotic protection under salt treatment and increased tolerance to salt stress (Navarro-León et al., 2021). Another example of employing the TILLING technique for obtaining a more salt tolerant crop comes from barrel clover (*Medicago truncatula*) (de Lorenzo et al., 2009). Two mutants carrying changes in *SRLK* (*Surface Receptor-like Protein Kinase*) gene showed insensitivity to the salt stress treatment in terms of root growth. This gene encodes a membrane-located receptor kinase, which plays an important role in stress signal transduction (de Lorenzo et al., 2009). In rice, nine genes associated with membrane transport – *OsAKT1 (Similar to AKT1-like potassium channel), OsHKT6 (Oryza sativa high-affinity K+ transporter), OsNSCC2 (Translocation protein Sec62 family protein), OsCAX2 (Sodium/calcium exchanger protein), OsHAK11 (Potassium transporter), OsP5CS1 (Delta-1-pyrroline-5-carboxylate synthetase), OsNHX (Sodium/hydrogen Exchanger), OsNAC60 (Similar to NAM/CUC2-like protein), OsSOS1 (Similar to Na+/H+ Antiporte*r) were subjected for TILLING analysis (Hwang et al., 2017). In total nine mutants carrying changes in five (*OsAKT1, OsHKT6, OsNSCC2, OsHAK11, OsSOS1*) of nine investigated genes exhibited higher tolerance to salt treatment compared to the wild type, in regards to root and shoot growth.

TILLING mutants have been employed also in studies related to plant response to heavy metals or to aluminum (Al^3+^) toxicity in acid soils. Decreased heavy metal accumulation in tobacco (*Nicotiana tabacum*) was achieved by TILLING analysis of two genes – *HMA2S* and *HMA4T*, which encode heavy metal transporters: cadmium (Cd) and zinc (Zn). They play a role in root-to-shoot metal transport. In the identified mutants, compared to the wild type, the levels of Cd and Zn in leaves decreased by 39-97% and 35-48%, respectively (Gao et al., 2020). The homolog of one of these genes – *HMA4a* was selected for TILLING analysis in yellow mustard (*B. rapa ssp. trilocularis*). The mutant with higher tolerance to Cd and Zn was identified, which may be used in Cd phytoremediation programs (Blasco et al., 2019; Navarro-Leon et al., 2019).

Aluminum is the most common metal in the Earth’s crust. In acidic soils, it solubilizes to phytotoxic Al^3+^ ions that cause root growth inhibition and may lead to yield reduction (Mossor-Pietraszewska, 2001; Bhalerao and Prabhu, 2013). Crop sensitivity to toxic Al^3+^ ions has become an important agronomic issue because of the progressive acidification of arable lands due to industrialization, acid rain, and the overuse of ammonia- and amide-containing fertilizers. It is known that Al^3+^ ions induce DNA damage and activate the DDR (DNA Damage Response) pathway, which leads to cell cycle inhibition and activation of DNA repair mechanisms (Nezames et al., 2012; Jaskowiak et al., 2018). TILLING analysis has been performed in barley for the *ATR* gene, which encodes kinase activated upon DNA damage that is involved in DDR signaling. Two of identified mutants show increased Al tolerance, resulting from disturbances in the DDR pathway leading to normal cell cycle progression despite DNA damage caused by Al^3+^ (Szurman-Zubrzycka et al., 2019b). In general, increasing Al tolerance in barley is a very important task due to the fact that it is the most Al-sensitive species among cereal crops (Ishikawa et al., 2000; Wang et al., 2006; Taylor and Greene, 2003; Szurman-Zubrzycka et al., 2021).

### 3.2 TILLING for improved product quality

Domestication and selection processes led to narrowing down the genetic variability for many crops. Nowadays, in breeding programs, it is necessary to reverse this process and TILLING-derived plant lines may possess desired genetic variability. Product quality is very important for consumers and, depending on the crop species, it may be related to various aspects including flavor and taste, health-promotion traits, sustainable production without the use of chemical inputs, quality, and nutritional content, or prolonged fruit shelf-life (Gascuel et al., 2017). There are many examples of using TILLING for improving product quality traits. In soybean (*Glycine max* L.), mutants in genes encoding four fatty acid desaturases that control saturated/unsaturated fatty acid ratio were isolated in a TILLING population that was used to decipher the oil biosynthesis pathway. Among these mutants, there were lines with improved seed oil composition – elevated oleic acid and lower polyunsaturated fatty acids contents (Lakhssassi et al., 2021a; Lakhssassi et al., 2021b). It should be noted that oils with increased levels of oleic acid are desired in the human diet. On the other hand, TILLING can be used for producing mutants with decreased content of undesired compounds or even free of them.

Low phytic acid mutants identification was a goal of Sashidhar’s and co-workers’ studies (2020) in oilseed rape (*Brassica napus* L.), a polyploid species. Phytic acid is the major phosphorous storage compound in seeds of this species (2-4%), but due to the fact that it cannot be metabolized, similarly to glucosinolates, tannins, and phenolic acids, it is considered to be an antinutritive compound (Thompson et al., 1990; Campbell et al., 2016). Researchers identified TILLING mutants carrying changes in two paralogs of *Bn2-PGK2 (2-Phosphoglyceric Acid Kinase)* genes. The double mutant for *Bn2-PGK2* had significantly reduced phytic acid contents, which is potentially beneficial for the human diet (Sashidhar et al., 2020).

Wheat is one of the most important crops worldwide, being a major source of energy and nutrition, but because of the high content of gluten, it cannot be eaten by some people with health issues (celiac disease). The TILLING strategy has been used to identify wheat genotypes with reduced content of immunogenic gluten proteins (Wen et al., 2022). Double mutants in durum wheat and triple mutants in common wheat with complete activity suppression of DME (DEMETER, 5-methylcytosine DNA glycosylase/lyase) and DRE2 (an iron-sulfur cluster biogenesis enzyme) displayed reduced content of immunogenic gluten proteins while retaining essential baking properties. Composition of starch is another quality trait that has been modified through TILLING in both common and durum wheat. The ratio of amylose:amylopectin influences the properties of starch. Resistant starch with higher amylose content is associated with health benefits. TILLING analysis in common wheat for two genes encoding starch synthases (SSIIa and SSIIIa) led to the identification of mutants with higher amylose content, with triple mutants (in A, B, and D genomes) having the strongest phenotypes (Schoen et al., 2021; Fahy et al., 2022).

Enrichment of provitamin A content in durum wheat (*Triticum durum*) grain is another example of the use of TILLING strategy to modify genes responsible for nutritional value. Vitamin A deficiency (VAD) is a public health problem in more than half of all countries, especially those in Africa and South-East Asia. The most severe effects of this deficiency are seen in young children and pregnant women in low-income countries. The transgenic approach to address VAD problem has been undertaken in rice (*Oryza sativa* L.) and genetically engineered ‘Golden Rice’ with enhanced provitamin A content was finally approved for cultivation in 2021 in The Philippines, after almost 20 years of studies (www.isaaa.org). In durum wheat, the increase of β-carotene content by more than 70% was achieved by TILLING of two genes, *HYD1 (β-carotene hydroxylase 1)* and *LcyE (Lycopene ϵ-cyclase)* (Sestili et al., 2019; Garcia Molina et al., 2021). Owing to the polyploid nature of durum wheat (4X), it was necessary to identify mutations in target genes located in both subgenomes (A and B).

A spectacular example of breeding success with the use of TILLING strategy also comes from wheat (in this case common wheat). For this species, tolerance to herbicide glyphosate (N-phosphonomethyl-glycine) was introduced through a nontransgenic approach (Moehs et al., 2021). The double wheat mutant, which carries missense mutations in A and D subgenomes in the gene *EPSPS* (*5-enolpyruvylshiki-mate-3-phosphate synthase*) showed enhanced tolerance to this herbicide (Green, 2018). Taking into account that glyphosate is the world’s most widely employed herbicide and genetically modified (GM) glyphosate-tolerant cultivars of maize, soybean and cotton have been widely used, the new sources of glyphosate tolerance may be very important for breeding. It should also be underlined that GM wheat tolerant to glyphosate (Roundup Ready™ wheat), developed by Monsanto in 2004, has never been cultivated in any country, including the USA (www.isaaa.org).

In tomato (*Solanum lycopersicum* L.), the improvement of fruit shelf-life has been a frequently studied trait, modified by TILLING for breeding purposes (Okabe et al., 2011; Okabe et al., 2012; Mubarok et al., 2015; Minoia et al., 2016; Brisou et al., 2021). One example is a study of Brisou et al. (2021) who identified mutants carrying changes in the *SlACO1 (ACC oxidase 1)* gene. The mutants exhibited decreased ethylene production and conductivity, which led to enhanced shelf-life and firmness. Another study aimed at the development of TILLING mutants with changed ethylene metabolism, shelf-life duration and fruit softening was performed for the *Ethylene Receptor* gene (*SlETR1*) (Mubarok et al., 2015). TILLING mutants with prolonged shelf-life were also identified through TILLING of *Expansin 1* (*SlExp1*) involved in cell wall expansions and loosening. The improvement of fruit shelf-life in these mutants was attributed to altered cell wall polysaccharide composition (Minoia et al., 2016).

## 4 Pros and cons of TILLING

The biggest advantage of TILLING, contrary to transgenic techniques, is that it is applicable to all species regardless of their genome size or transformation potential. There is no need to use sophisticated tissue cultures that for some species or genotypes are impossible to maintain. After traditional mutagenesis used for the creation of TILLING population, mutations can be found in every gene of interest. In general, the higher density of mutations the smaller population is needed to be analyzed to find mutations within each gene. With the use of TILLING one can identify the series of alleles (potentially giving “weak” and “strong” phenotypes), not only knock-outs. It is of special importance, especially for genes that are crucial for plants to survive, whose total inactivation may be lethal. For them, the sublethal alleles may be required for phenotypic analysis. Advantage from the reduction of protein activity obtained with TILLING-mutants vs. total turn off of the gene function using RNAi technique can rely also on avoiding some disruption during plant growth and development, which was indicated e.g. in research in tobacco, where RNAi mutants were characterized by necrotic lesions or increased water content in leaves (Liedschulte et al., 2017). Importantly, the analysis of multiple “weak” and “strong” alleles allows the deepening of knowledge on gene function. In barley analysis of the *HvHox1* (*homeodomain-leucine-zipper1*) gene, which controls the row-type character of a spike, showed that three forms carrying missense mutation presented different phenotypes compared with the parent cultivar ‘Barke’. It reflects the different influences of substitutions on the protein function. Two mutants presented intermediate phenotypes, another one a six-row type spike, while cultivar ‘Barke’ was two-rowed (Gottwald et al., 2009). Another example of an increase in knowledge about gene function comes from Arabidopsis and mutant generated by TILLING in *ABP* (*Auxin Binding Protein 1*) gene - *abp1-5* (Henikoff et al., 2004). This mutant carries a missense mutation leading to substitution in the auxin binding pocket of ABP1, which might alter the auxin binding process. Studies using the TILLING mutant led to the revealing of many auxin-related roles for ABP1, while null alleles, appeared to be embryo-lethal (Enders et al., 2015). TILLING could be also the technique of choice when complete loss of function of the investigated gene is undesired in breeding programs. An example of the necessity to obtain so-called “weak” alleles (that can be provided by TILLING) was shown e.g. in the study of Liedschulte et al. (2017) on the reduction of cadmium in leaves of tobacco (*Nicotiana tabacum*). The low level of Cd might be „beneficial” for smokers because it leads to lower Cd accumulation in their bodies. The study conducted on inhabitants of the Upper Silesia region in Poland indicated that smokers accumulated a twice higher level of Cd compared with non-smokers (Bem et al., 1993). First, two heavy metal transporter homeologous genes - *HMA4.1* and *HMA4.2* were silenced using an RNAi (RNA interference) approach. This was successful for Cd reduction in tobacco leaves, however negative effects on plant development, including retarded growth, necrotic lesions, altered leaf morphology, and increased water content have been observed. Application of the TILLING strategy led to removing this impact, and mutants with both, a lower content of Cd in leaves and no negative developmental effects have been identified. In order to minimize the phenotypic effects, the different mutation combinations in analyzed genes have been investigated. It has been proven, that complete functional loss obtained by induction of nonsense mutation in one homeologous *HMA4* gene and the functional reduction in the other *HMA4* gene obtained by induction of missense mutation led to the best results and these mutation combinations are the best choice for breeding programs in tobacco (Liedschulte et al., 2017).

The TILLING strategy has been used for more than 20 years, during which it was modified and improved. In general, as described in previous sections, the screening methods keep getting better, faster, and cheaper, which makes TILLING a robust tool for functional genomics. This can be seen especially in terms of sequencing technology, but currently, the use of TILLING-by-sequencing is limited to species for which reference genomes are available. The software for mutation analysis is also evolving together with TILLING. As an example, the PARSESNP tool (Taylor and Greene, 2003) was very useful at the beginning of TILLING research, however, it is not active anymore, whereas new tools are arising e.g. Mutation Finder Annotator (https://github.com/bjtill/Mutation-Finder-Annotator-CLI) that can be used for TILLING by sequencing. It is noteworthy that TILLING can also be conducted without any sophisticated apparatus, utilizing only basic laboratory equipment (e.g. with product visualization on agarose gel). Such a low-cost approach is suitable for implementation in developing countries with limited laboratory infrastructure, however, it is important to acknowledge that it may result in reduced sensitivity when it comes to identifying mutations.

A significant advantage of TILLING is that once established, a TILLING platform is long-lasting and may be used for both, forward and reverse screening. Finally, most important from an agronomic point of view, non-GMO mutants are created, as in TILLING strategy there is no need for transformation. Hence, new TILLING alleles may be used not only for functional analysis of genes but also serve as a valuable resource for crop improvement because they can be directly used in breeding programs without regulatory restrictions that exist in some countries for the material labeled as GMO.

TILLING, like any other approach, has also some limitations. Establishing TILLING platform may be time- and labor-consuming, but once established it may serve as a source of mutation for many years. However, the biggest disadvantage of TILLING is, without a doubt, the presence of background mutations that can affect the phenotype and, hence, impede gene function analysis. It was shown that in an *abp1-5* mutant carrying change in the *ABP* (*Auxin Binding Protein 1*) gene of Arabidopsis, which was identified by TILLING approach, after whole-genome sequencing the additional 8,000 single nucleotide polymorphisms were identified (Enders et al., 2015). It was also calculated that for barley, in a TILLING population with mutational density of 1/500 kb, there are ca. 10,000 mutations in the genome of each plant (Szurman-Zubrzycka et al., 2018). However, it has to be noted that the coding part of a genome is usually very small (in the case of barley, it is 1.4%; Mascher et al., 2017), so most of these 10,000 mutations are in the noncoding sequences, with the lower probability of impacting the phenotype. What is more, some of those in coding regions may be silent or cause amino-acid substitutions that do not affect the protein activity. Taking it all into account, the total number of background mutations that was problematic in terms of functional analysis is low enough to be removed from the background by backcrosses with the parent variety. One backcross of a TILLING mutant with a parent variety reduces the number of background mutations by half, and performing a higher number of backcrosses can be recommended. However, it should be kept in mind that multiple backcrossing will prolong the time before phenotyping. The F_1_ generation is heterozygous in terms of analyzed mutation. The homozygous mutants should be selected from the BCF_2_ generation. Mutations are very often recessive, which means that only individuals carrying a mutation in a homozygous state should be phenotyped (Okabe et al., 2011). Such backcrossing is often time-consuming and the high density of background mutations is considered to be the main problem in the application of TILLING strategy as a reverse genetic tool. This problem is avoided in the modern targeted genome editing methods, such as CRISPR/Cas9 (reviewed in Gaj et al, 2013; Mei et al., 2016; Liu et al., 2021) and TALEN (reviewed in Becker and Boch, 2021), which are two powerful genome editing (GE) tools that have revolutionized plant biotechnology. In the case of these GE methods, called also New Genomic Techniques (NGTs), the mutation can be introduced specifically to the target position within the gene of interest, and the probability of off-target mutation appearance is relatively low. It is the main advantage of CRISPR/Cas9 and TALEN over TILLING. Both, CRISPR/Cas9 and TALENs have been successfully employed in plant research, and being the precise and efficient methods for modifying plant genomes, they have opened up new possibilities for plant breeding and biotechnology. It has to be kept in mind, that in some countries materials produced through these technologies are labeled as GMOs, even though the stable CRISPR/Cas9 or TALEN mutants do not carry any transgene and do not possess foreign DNA. However, the regulatory landscape in this regard is continuously evolving, and the legal status of CRISPR/Cas9 mutants is undergoing changes, reflecting the shifting perspectives and emerging legislative frameworks in various jurisdictions (e.g. the newest, dated July 5th 2023, proposal, for a REGULATION OF THE EUROPEAN PARLIAMENT AND OF THE COUNCIL on plants obtained by certain new genomic techniques and their food and feed, and amending Regulation (EU) 2017/625).

In general, TILLING and GE approaches should complement each other in functional genetic studies or in pre-breeding programs. There are several examples of joined usage of these approaches to study gene function. For example, Wang and co-workers by using CRISPR/Cas9 and TILLING strategies demonstrated that *TaGW2* homoeologs are negative regulators of grain size and weight, that contribute additively to the phenotype. The obtained TILLING and CRISPR/Cas9 mutants provide the opportunity to combine mutant alleles in various configurations, enabling fine-tuning of the phenotypic outcomes (Wang et al., 2018). However, it is also needed to be highlighted that for many plant species, including those agronomically important, there is no efficient protocol for transformation. Hence, GE technologies may be applied only to a limited number of species or to a limited number of cultivars of particular species (e.g. a protocol of barley transformation is efficient only for one cultivar - ‘Golden Promise’), whereas TILLING may be applied to any plant species and genotype.

The problem of background mutation was also reduced in a FIND-IT approach, because of using a mutagenized population with very low mutation density (Knudsen et al., 2022). This is a powerful barley platform generated with the use of NaN_3_, possessing high capacity for the detection of premature stop codons, which was demonstrated by the identification of one hundred such changes in genes of interest. Mutations that cause the emergence of a premature stop codon have a high probability of disrupting the function of the encoded protein. However, these types of mutations are detected with low frequency as compared with other types of mutations (silent, missense) in previously reported plant TILLING populations (Kurowska et al., 2011; Szurman-Zubrzycka et al., 2018, Gao et al., 2020; Brisou et al., 2021).

## 5 Concluding remarks

We are currently in the so-called postgenomic era. Due to the high throughput methods of genome sequencing, there is more information about sequences than about gene function. Functional analysis of genes lags behind the acquisition of new sequences of whole genomes. It is well established that induced mutations have been a powerful tool in functional genomics and breeding for over 80 years of their use in plant research. Establishing a TILLING platform may be time-consuming, however for many species, these platforms have already been developed and the identification of TILLING mutants is really fast. Additionally, one can predict that as technologies of mutation detection are getting better and faster, so TILLING will too. As an example, FIND-IT, the new TILLING-based approach, provides ultrafast screening for variants of interest (within 10 days). The bottleneck is usually the characterization of identified mutants – determining the phenotypic consequence of the mutation, but this is a problem of each mutation technology, including gene editing by CRISPR/Cas9. In terms of functional genetics studies, it is always an added value to combine different approaches that complement each other, e. g. TILLING and CRISPR/Cas9 strategies that produce different types of mutants. It is also worth reminding that TILLING mutants are not GMOs so they can be directly used, without regulations, in pre-breeding programs around the world to improve crop performance.

## Author contributions

MS-Z and IS conceive the manuscript. MS-Z and MK wrote the manuscript; MK generated tables; BT and IS revised and edited manuscript. All authors approved the submitted version.

## Glossary

**Table:**

ABA	Abscisic Acid
*ABP*	Auxin Binding Protein 1
ATR	Ataxia Telangiectasia and Rad3-related
Bn2-PGK	Brassica napus 2-Phosphoglyceric Acid Kinase; bZIP, Basic Leucine Zipper
CEL I	Celery Endonuclease I
CRISPR/Cas9	Clustered Regularly Interspaced Short Palindromic Repeats/CRISPR-associated protein 9
ddPCR	Droplet Digital PCR
DDR	DNA Damage Response
DHPLC	Denaturing High-Performance Liquid Chromatography
EMS	Ethyl MethaneSulphonate
*EPSPS*	5-enolpyruvylshiki-mate-3-phosphate synthase; FIND-IT, Fast Identification of Nucleotide variants by droplet DigITal PCR
GE	Genome Editing
GMO	Genetically Modified Organism
HRM	High-Resolution Melting
*HvABI5*	Hordeum vulgare ABA Insensitive 5
HvCBP20	Hordeum vulgare Cap-Binding Protein 20
HvERA1	Hordeum vulgare Enhanced Response to ABA 1
HvHox1	Homeodomain-leucine-zipper 1
HYD1	β-carotene hydroxylase 1; IPCC, Intergovernmental Panel on Climate Change
*LcyE*	Lycopene ϵ-cyclase; NGS, Next-Generation Sequencing
NGTs	New Genomic Techniques
*OsAKT1*	Oryza sativa AKT1-like Potassium Channel
OsCAX2	Oryza sativa Sodium/Calcium Exchanger Protein
OsHAK11	Oryza sativa Potassium Transporter
OsHKT6	Oryza sativa High-affinity K+ Transporter
OsNAC60	Oryza sativa NAM/CUC2-like Protein
OsNHX	Oryza sativa Sodium/Hydrogen Exchanger
OsNSCC2	Oryza sativa Translocation Protein Sec62 Family Protein
OsP5CS1	Oryza sativa Delta-1-pyrroline-5-carboxylate Synthetase
OsSOS1	Oryza sativa Na+/H+ Antiporter; RNAi, RNA interference
RWC	Relative Water Content
SDGs	Sustainable Development Goals
*SlACO1*	Solanum lycopersicum ACC Oxidase 1
SRLK	Surface Receptor-like Protein Kinase; TALEN, Transcription Activator-Like Effector Nucleases
TILLING	Targeting Induced Local Lesion IN Genomes
VAD	Vitamin A Deficiency
